# Pathogenetic Therapy of Psoriasis by Muramyl Peptide

**DOI:** 10.3389/fimmu.2019.01275

**Published:** 2019-06-20

**Authors:** Svetlana Guryanova, Vladislav Udzhukhu, Aleksandr Kubylinsky

**Affiliations:** ^1^Shemyakin–Ovchinnikov Institute of Bioorganic Chemistry, RAS, Moscow, Russia; ^2^Medical Institute, RUDN University, Moscow, Russia; ^3^AO Peptek, Moscow, Russia; ^4^Pirogov Russian National Research Medical University (RNRMU), Moscow, Russia

**Keywords:** psoriasis, muramyl peptide, sCD54, MIF, IL-4, IL-10, IL-12, TNF-α

## Abstract

Psoriasis is a multifactorial disease with a dysregulation in immune system. The aim of this study was to survey the clinical efficacy and safety of muramyl peptide—the ligand of the receptors of innate immunity (drug Licopid, AO Peptek, Moscow, Russia) in patients with psoriasis. The effect of muramyl peptide on 86 patients with different severity of plaque psoriasis was tested. The Psoriasis Area and Severity Index (PASI), cytokine status and production of nitric oxide in blood serum, and the subsequent course of psoriasis have been evaluated. Evaluation of significance of observed differences was presented by the Student's *t*-test. As a result of the treatment, clinical cure or improvement was detected in 98.2% of patients (*p* < 0.05), while 24.4% had a complete cure. Subsequent observations during 4 years showed that patients who received muramyl peptide statistically significantly increased relapse-free period. Moreover, subsequent relapses of the disease after treatment with muramyl peptide were in much more milder form in the cases of mild psoriasis. The conducted studies showed that monotherapy with muramyl peptide stopped the clinical manifestations of psoriasis, normalized the processes of cytokine-dependent [interleukin (IL)−4, IL-10, IL-12, tumor necrosis factor alpha (TNF-α)] regulation of the immune response and nonspecific resistance, expressed in a decreasing amount of serum antigens sCD54 [soluble intercellular adhesion molecule-1 (sICAM-1)] to reference values (*p* ≤ 0.01). Taken together, our research demonstrated the effectiveness of therapy with muramyl peptide and moreover, that elevated levels of sCD54 and MIF (*p* ≤ 0.01) in the serum of patients with psoriasis considered as potential biomarkers of the severityof psoriasis and control over their dynamics have prognostic significance in determining the effectiveness of the therapy.

## Introduction

Psoriasis is a chronic, noncommunicable, painful, disfiguring, and disabling disease for which there is no cure with negatively impact clinical outcomes and quality of life (1). The urgency of studying the mechanisms of psoriasis development and the investigation of new methods of treatment is due to its wide prevalence in developed countries: from 3.2% in USA (2, 3) till 11.9% in Norway (4) and significant resistance to standard methods of treatment (5, 6). Currently, it is customary to consider psoriasis as a disease in which genetic control and metabolic disorders occur (7–9) and systemic therapy is required (10, 11). Often there are persistent imbalances in the immune system and pathological changes in the microcirculatory stream (12, 13). It is assumed that the dysfunctional state of immune homeostasis in patients with psoriasis, largely due to the inability of immunocompetent cells to respond adequately to signals coming in during the immune response, including suppression (14, 15), particularly inhibiting keratinocyte proliferation (16, 17). It should be noted that the psoriatic process is most sensitive to immunosuppressive effects (methotrexate, cyclosporine, psoralen and ultraviolet A (PUVA) therapy, biologically active drugs). However, such therapies are often accompanied by unwanted side effects, often leading to more severe clinical relapses, which significantly limit the range of their use (18–20). The emergence of a persistent imbalance in the immune system underlying the cellular-molecular mechanism of the development of pathological processes in psoriasis has created the prerequisites for the development of treatment methods that can effectively affect the pathogenesis of the disease and thereby lead to healing. Thus, the use of drugs that can target specific receptors of innate immunity, such as muramyl peptide (MP), is pathogenetically substantiated in the treatment of psoriasis.

Muramyl peptides are the component of bacterial cell walls and modulate through Nucleotide-binding oligomerization domain-containing protein 2 (NOD2) the function of innate and adaptive immunity (21, 22). The role of MP in the induction of the inflammatory process has been well studied. *In vitro* activation of Nod2 leads to transcription of multiple genes, often mediated through NF-κB activation and mitogen-activated protein kinas signaling, which results in the production of pro-inflammatory mediators and antimicrobial molecules (23, 24). At the same time, there are also data on the negative feedback on inflammation of the NOD-2-mediated immunity (25, 26). NOD2 seems to have a dichotomous function being involved both in pro- and anti-inflammatory responses to microbial stimuli. Muramyl peptides more than 20 years are widely used in medical practice to correct immunopathological processes (27, 28), including psoriasis (29). Williamson et al. (Department of Dermatology, University Hospital of Wales and University of Wales College of Medicine, Cardiff, UK) treated with glucosaminyl muramyl peptide 10 patients with stable, chronic plaque psoriasis. “Patients were given 20 mg of oral GMDP daily for 14 days. Severity of psoriasis was assessed at baseline, 3, 7, and 14 days by the psoriasis area and severity index (PASI) score, and plaque thickness was determined by pulsed A-scan ultrasound. After treatment with GMDP, results showed significant change in PASI score with time and there was a marked improvement in the psoriasis of all 10 patients” (29).

The purpose of this study was to study the clinical efficacy and safety of the muramyl peptide in patients with psoriasis, its effect on the cytokine status and production of nitric oxide, and the subsequent course of psoriasis. The limitation of presented investigation rests in the comparison of cytokine levels in serum of patients with psoriasis before and after treatment by muramyl dipeptide with those of healthy donors and lack of comparison with a placebo-treated group of patients with psoriasis.

## Patients and Methods

### Ethic Statement

This study was carried out in accordance with the manufacturer's instructions for treatment psoriasis (Registered with the Ministry of Health, Russian Federation). Protocol was approved by the Ethics Committee of the Pirogov's Russian National Research Medical University (Moscow, Russia). All subjects gave written informed consent in accordance with the Declaration of Helsinki.

### Patients

A total of 86 patients (50 women and 36 men aged 19–63 years) received the total treatment (outpatient and inpatient). The duration of the disease varied from 6 months to 45 years. Among the provoking factors, psycho-emotional stress, exacerbations of chronic diseases, acute respiratory viral infections were most often observed. The frequency of relapses averaged 2–3 per year. The study of the structure of concomitant diseases did not reveal any peculiarities in comparison with the average statistical distribution in the European part of the Russian Federation. Most often, the patients examined had lymphopenia accelerated by the erythrocyte sedimentation rate (ESR).

**Figure 1:**
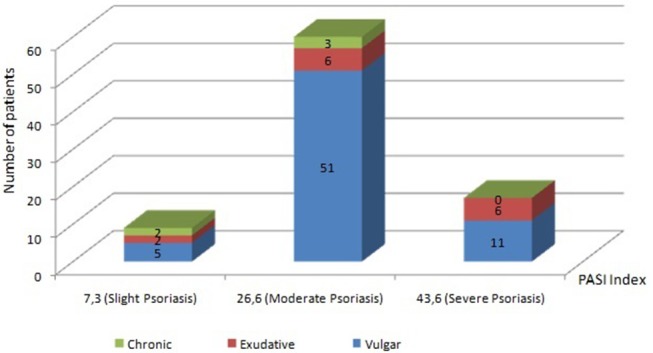
Patients' distribution by types and severity of psoriasis. The vertical scale and the numerical values in the figure represent Number of patients. Slight degree of severity of the disease course was detected in 9 patients (PASI = 7.3 ± 1.8). Among them, 5 have vulgar psoriasis, 2 have exudative and 2 chronic forms of psoriasis. In 60 patients (51 with vulgar, 6 with exudative and 3 with chronic psoriasis) with moderate severity of psoriasis, the mean PASI was 26.6 ± 4.2. In 17 patients (11 patients with vulgar and 6 with exudative psoriasis) with a severe course of the skin-inflammatory process, the mean PASI value was 43.6 ± 3.9.

A vulgar variety of psoriasis is diagnosed in 67 people, represented by various plaques and papules of pink color with silver-white scales on the surface. In 14 patients, during the initial examination, an exudative variety of the disease was revealed, at which the phenomena of pronounced secondary wetness were observed. In 5 patients, a chronic form of the disease characterized by long-standing plaques with significant papular infiltration was defined. In the majority of patients, psoriasis was in a progressive stage, flowing as an undifferentiated type of disease. The PASI index was used as a tool for determining the severity of the course of the pathological process and the effectiveness of the therapeutic measures performed. In accordance with the obtained values of PASI index, slight degree of severity of the disease course was detected in 9 patients (PASI = 7.3 ± 1.8). Among them, 5 have vulgar psoriasis, 2 have exudative and 2 chronic forms of psoriasis. In 60 patients (51 with vulgar, 6 with exudative and 3 with chronic psoriasis) with moderate severity of psoriasis, the mean PASI was 26.6 ± 4.2. In 17 patients (11 patients with vulgar and 6 with exudative psoriasis) with a severe course of the skin-inflammatory process, the mean PASI value was 43.6 ± 3.9. Patients were treated without “blinding” and untreated or placebo-treated patients were not tested. The distribution of patients according to the type and severity of psoriasis is presented in Figure 1.

### Biomarkers and Nitric Oxide Quantification

The level of cytokines and soluble macrophage migration inhibitory factor (MIF) was determined in the blood serum of patients with psoriasis by enzyme-linked immunosorbent assay (ELISA) using Pro-con kits (Protein Contour LLC, St. Petersburg, Russia). The content of soluble antigens CD54 (sCD54, sICAM-1) was determined by an enzyme immunoassay using the Human sICAM-1/CD54 ELISA kit to determine sICAM-1 levels in serum (R&D Systems, Minneapolis, USA). The detection limit was 1.56 ng/mL.

Quantitative analysis of the content of nitric oxide (NO) in the blood serum was assessed by the level of its stable metabolites—NO_2_, NO_3_ ions, determined using a spectrophotometer DU-50 (Beckman, USA) at a wavelength of 520 nm.

As a control, reference values were used, corresponding to the average statistical values of the studies that were established in the laboratory after examination of 50 practically healthy donors aged 18–60 years.

### Drug Administration

In the treatment of all patients with psoriasis, a medicine based on N-acetylglucosaminyl-N-acetylmuramyldipeptide (GMDP, drug Licopid^®^, AO Peptek, Moscow, Russia) was used for 1 tablet (10 mg) for half an hour before meals 2 times a day during 20 days.

According to the indications (severe itching, burning), H1-blockers were additionally prescribed (cetirizine 10 mg per oz/day for 7 days). In a number of cases (2 women with exudative psoriasis), during the first 7 days of treatment, topical steroids (cream and 0.1% hydrocortisone emulsion) were used that were applied to individual lesions in the area of the facial skin and open areas of the upper limbs.

### Statistical Analysis

Statistical analysis and graphical illustration of numerical data was performed using Graph Pad Prism (GraphPad Software Inc.). Data are presented as mean ± SD, SEM. Statistical significance was determined by Student's *t*-test. *P* < 0.05 was defined as statistically significant ^*^p < 0.05, ^**^p ≤ 0.01.

## Results and Discussion

The evaluation of the effectiveness of the therapy with muramyl peptide was carried out according to generally accepted criteria (30). The majority of patients showed positive dynamics during the pathological process from the first days of application of the muramyl peptide. Unpleasant subjective sensations in the form of itching, burning, feeling of tight skin in the field of psoriatic plaques were stopped. By the 8th−10th day of application of the muramyl peptide preparation, in most patients there was a cessation of the appearance of fresh rashes, a significant decrease in the severity of the inflammatory phenomena, the intensity of peeling and secondary wetness, and the disappearance of the peripheral rim of growth in the area of the affected skin. Subsequently, the epithelization of surface and deep cracks occurred, a marked decrease in infiltration, a reduction in the area of the affected skin, and then a complete or partial resolution of the pathological process.

The evaluation of the effectiveness of the therapy with muramyl peptide was carried out according to generally accepted criteria. The disappearance of all clinical signs and symptoms compared with the baseline (improvement of the PASI index by 100%) was regarded as a clinical cure. Reduction in the severity of all clinical signs and symptoms compared with the baseline, in addition to residual effects in the form of mild erythema (improvement in the PASI index of 75% ≤PASI≥99%) was defined as a significant improvement. Regression of clinical symptoms of psoriasis in the form of an improvement in the PASI index of 50% ≤PASI≥74% was regarded as an improvement. Reducing the intensity of all clinical signs and symptoms compared to the baseline as an improvement in the PASI index of 25% ≤PASI≥49% was considered to be a satisfactory improvement. The improvement in PASI was <24% regarded as a minor improvement. In cases of lack of dynamics from clinical symptoms or worsening during psoriasis, it was assessed, respectively, as unchanged or worsening.

**Figure 2:**
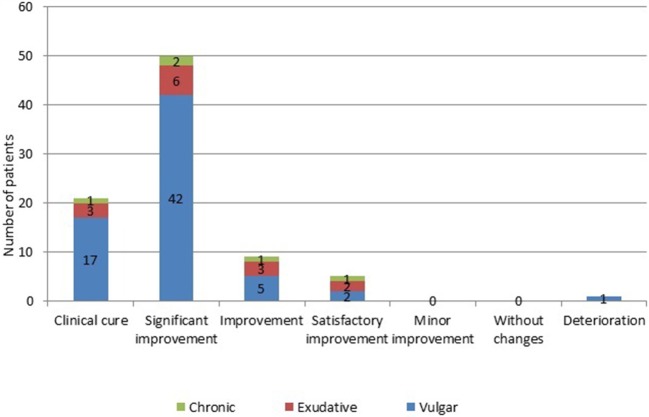
Number of patients with psoriasis evaluated on the effectiveness of the treatment with muramyl peptide. The vertical scale and the numerical values in the figure represent Number of patients. A significant improvement was noted in 50 (58.1%) patients (42–with vulgar, 6–with exudative, and 2–with chronic psoriasis). Improvement of all clinical signs and symptoms in comparison with the initial condition was characteristic for 9 (10.4%) patients (5–with vulgar, 3–with exudative, and 1–with chronic psoriasis). Only 5 (5.9%) patients (in 2 with vulgar, 2 with exudative, and 1 with chronic psoriasis), the results of therapy were defined as satisfactory improvement. Deterioration during the pathological process was observed in 1 (1.2%) of a patient with a vulgar variety of a disease who had previously received systemic treatment with methotrexate.

In 21 (24.4%) patients (17–with vulgar, 3–with exudative, and 1–with chronic psoriasis) after the treatment, there was a clinical cure, consisting in complete absence of manifestations of the pathological process (Figure 2). A significant improvement was noted in 50 (58.1%) patients (42–with vulgar, 6–with exudative, and 2–with chronic psoriasis). Improvement of all clinical signs and symptoms in comparison with the initial condition was characteristic for 9 (10.4%) patients (5–with vulgar, 3–with exudative, and 1–with chronic psoriasis). Only 5 (5.9%) patients (in 2 with vulgar, 2 with exudative and 1 with chronic psoriasis), the results of therapy were defined as satisfactory improvement. Deterioration during the pathological process was observed in 1 (1.2%) of a patient with a vulgar variety of a disease who had previously received systemic treatment with methotrexate.

**Figure 3:**
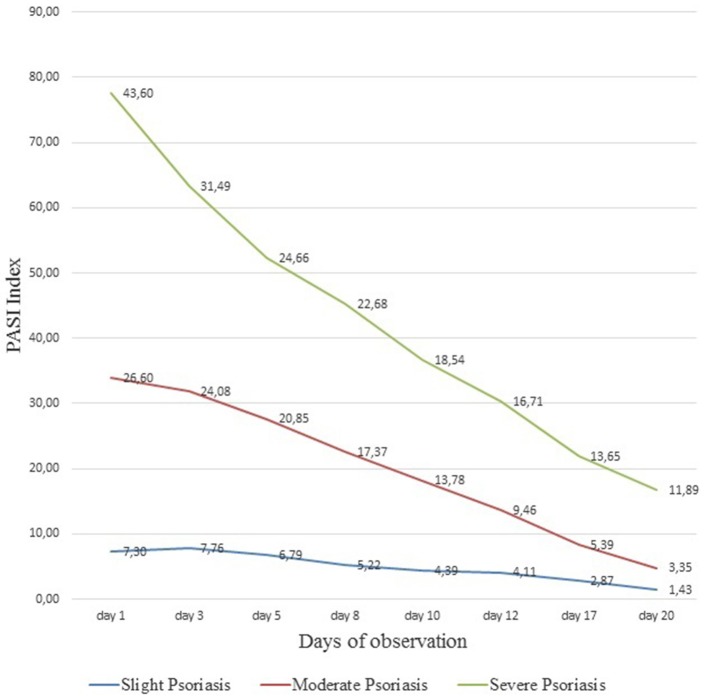
Dynamics of PASI index in the course of ongoing therapy with muramyl peptide. The analysis of the conducted studies showed that in patients with severe psoriasis the PASI index decreased from 43.6 ± 3.1 to 11.89 ± 1.5, with an moderate degree from 26.6 ± 2.2 to 3.35 ± 2, 4, with a slight degree of severity of psoriasis—from 7.3 ± 2.49 to 1.43 ± 0.6. Statistical significance in all measurements *p* < 0.05.

The analysis of the conducted studies showed that in patients with severe psoriasis the PASI index decreased from 43.6 ± 3.1 to 11.89 ± 1.5 (*p* < 0.01), with an moderate degree from 26.6 ± 2.2 to 3.35 ± 2.4 (*p* < 0.01), with a slight degree of severity of psoriasis–from 7.3 ± 2.49 to 1.43 ± 0.6 (*p* = 0.037). Statistical significance in all measurements was *p* < 0.05 (Figure 3). Dynamic observations in patients with varying severity of psoriasis during the course of the therapy did not reveal any side effects and complications in the course of the therapy. The results of subsequent observations in the time interval from 6 months to 4 years showed that patients who received muramyl peptide statistically significantly (more than twice) increased relapse-free period (relapses of psoriasis were observed on average 1 time per year and before treatment of glucosaminyl muramyl dipeptide 2–3 times a year). It is important to note that subsequent relapses of the disease were much milder in the form of mild severity of psoriasis.

Thus, according to the principles of modern evidence-based medicine, in terms of the level of reliability, muramyl peptide in psoriasis can be attributed with good reason to drugs characterized by A level efficacy (high efficacy in 80–100% of patients).

**Figure 4:**
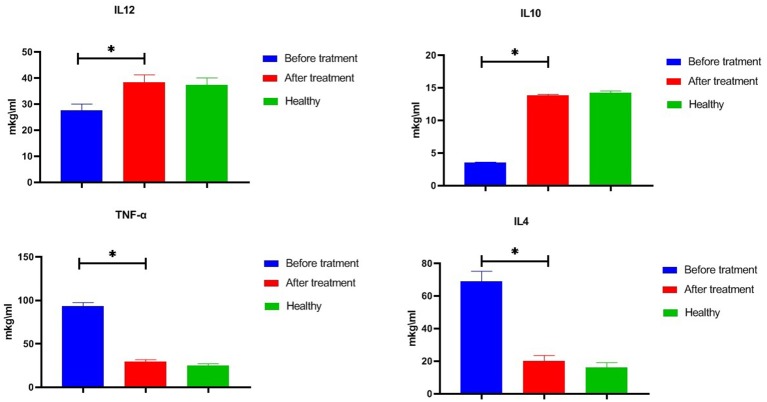
Effect of glucosaminyl muramyl peptide on the serum cytokine production of patients with psoriasis. Cytokine production 86 patients with psoriasis before and after treatment with glucosaminyl muramyl peptide in comparison with cytokine production of 50 practically healthy donors aged 18–60 years. Therapy with muramyl peptide medication contributed to the normalization of the cytokine levels. Data are shown as mean ± SEM. Statistical significance *p < 0.05.

Studies conducted in 86 patients with psoriasis (Figure 4) revealed serious imbalances in the state of the cytokine system relative to the level of cytokines 50 practically healthy donors aged 18–60 years. Particularly, in the serum of peripheral blood the concentrations of IL-12 (up to 27.6 ± 2.4 pg/ml) were significantly lower compared with the reference values (37.4 ± 2.6 pg/ml). A similar decrease in the level of IL-10 (up to 3.5 ± 0.1 pg/ml at reference values of 14.1 ± 0.2 pg/ml) was also determined. At the same time, there was a significant increase in the TNF-α vascular bed up to 93.4 ± 3.7 pg/ml and IL-4 to 69.1 ± 2.8 pg/ml at reference values of 25.0 ± 2.0 pg/ml and 16.1 ± 1.3 pg/ml. The use of glucosaminyl muramyl dipeptide significantly increased the content of peripheral blood IL-10 to 13.8 ± 0.9 pg/ml (*p* < 0.05), which in turn led to a decrease in the production of pro-inflammatory cytokines. Thus, the concentration in the vascular channel of TNF-α dropped from 29.6 ± 2.1 pg/ml (*p* < 0.05), and IL-4—to 17.5 ± 1.4 pg/ml (*p* < 0.05). After the treatment with glucosaminyl muramyl dipeptide, the blood content of IL-12 increased significantly to 38.4 ± 2.8 pg/ml (*p* < 0.05). Increased production of IL-12 led to the restoration of an adequate level of natural resistance and normalization of the functional activity of monocytic-macrophagal cells. Thus, therapy with muramyl peptide medication contributed to the normalization of the cytokine-dependent regulatory response and cellular internalization processes. Considering that the factor of inhibition of migration of macrophages (MIF) is a key regulator, acting at the initial stages of the development of the innate and acquired immune response, it seemed to us expedient to study the effect on its production of the drug muramyl peptide. The conducted studies showed that the concentration of MIF in the blood (31.5 ± 3.6 ng/ml, *p* ≤ 0.01) significantly increased (Table 1) in comparison with the reference values under the influence of the muramyl peptide drug decreased to the level of the parameters in healthy donors (5.9 ± 0.7 ng/ml). Thus, the use of muramyl peptide significantly reduced the ability of the MIF to induce the entry of macrophages and neutrophilic leukocytes into the psoriatic foci of inflammation and proliferation, as well as stimulate the secretion of TNF-α and IL-1β. Complex immunological studies made it possible to identify pathological shifts in the system of adhesion antigens in patients with psoriasis, characterized by a decrease in the concentration of soluble adhesion molecules (sCD54) and membrane forms of these antigens. The use of the muramyl peptide allowed eliminating imbalances in this important system of immunity. So, after the treatment in the peripheral blood of patients with psoriasis, there was a decrease of the level of serum antigens sCD54 from 266.1 ± 16.0 ng/ml to 186.1 ± 14.3 ng/ml (*p* ≤ 0.01), practically did not differ from similar values in healthy donors. Thus, the decrease of soluble adhesion molecules in the blood allowed stabilizing the activation processes of immunocompetent cells and cell migration. Studies have shown that the presence of inflammatory and functional changes in psoriasis led to an increase in the production of endogenous nitric oxide to 34.1 ± 1.5 μmol/L at 19.4 ± 1.4 μmol/L in healthy donors. A significant increase in nitric oxide compared with the reference values, passing the framework of protective immunity, contributed to the development of inflammatory phenomena in patients with psoriasis. After the therapy with muramyl peptide, the level of nitric oxide in the peripheral blood decreased to reference values [19.9 ± 1.3 μmol/L, (*p* ≤ 0.01)], which indicated the stabilization of immune metabolic processes (Table 1).

**Table 1:** Dynamics of inflammatory mediators in the course of ongoing therapy with muramyl peptide in peripheral blood.

**Groups** **(Number of patients)**	**MIF** **(ng/ml)**	**sCD54** **(ng/ml)**	**NO** **(μmol/L)**
Before treatment (*N* = 86)	31.5 ± 3.6^**^	266.1 ± 16.0^**^	34.1 ± 1.5^**^
After treatment (*N* = 86)	5.9 ± 0.7^**^	186.1 ± 14.3^**^	19.9 ± 1.3^**^
Healthy donors (*N* = 50)	5.5 ± 0.6	162.1 ± 10.4	19.4 ± 1.4

*Significance was calculated by using the t-test comparing the levels of inflammatory mediators before and after treatment with muramyl peptide*.

** *p ≤ 0.01*.

Treatment the psoriasis patients with glucosaminyl muramyl dipeptide 10 mg twice a day during 20 days promoted statistically significant change in clinical symptoms and in cytokine levels. It should be noted that the dominant group was the vulgar type severe form of the disease and it was in this group that the greatest positive clinical effect of muramyl peptide therapy was observed. The group with chronic psoriasis was the smallest, all patients in this group showed improvement. The obtained data on the clinical efficacy of muramyl peptides in the treatment of psoriasis are consistent with the results of other studies of patients with stable, chronic plaque psoriasis (29) in the significant decrease of PASI index. However, for greater reliability and statistical significance, it is necessary to conduct similar studies on a larger sample of patients.

Elevated levels of sCD54 and MIF in the serum of patients with psoriasis and control over their dynamics have prognostic significance in determining both the severity of the disease and the effectiveness of the therapy. The obtained data are consistent with the results of other researchers, indicating the value of serum markers sCD54 (31) and MIF (32, 33) in the pathogenesis of psoriasis, and can be considered as potential biomarkers and targets for immunomodulating therapies for psoriasis. Standardized serum markers measurement is available for clinical use and analysis of secreted protein after treatment by immunomodulators would offer a prognostic factor of the therapy effectiveness. Randomized and controlled clinical trials would be necessary to evaluate such a potential biomarker before implementation in clinical practice.

The mechanism of protective activity of glucosaminyl muramyl dipeptide is not fully defined. It is well established the influence of MP in induction of inflammatory processes. The anti-inflammatory properties of muramyl peptides are not widely discussed, although it is known that muramyl dipeptides limit infection and inflammation in murine model of sepsis (34), via NOD2 reduces fat inflammation (35). Recently more and more evidence that NOD2 stimulation activates a cross-tolerance response that downregulates and prevents excessive TLR responses has appeared. Chronic stimulation of NOD2 by muramyl dipeptide mediates tolerance to bacterial products (36) and enhances tolerance to enteric pathogens (37). One of the possible mechanisms that explain the emergence of tolerance is the ubiquitination and degradation of the NOD2 receptor, responsible for inflammation and manifestation of the pathological process (38). The found relationship between the severity of psoriasis and Crohn's disease, which appears as a result of a mutation in the NOD2 protein, confirms the validity of using muramyl peptides for the pathogenetic therapy of psoriasis (39). The mechanisms triggered by muramyl peptides, in particular, the shift in the balance of T-cell subpopulations (28) and regulating subpopulations of CD4+ and CD8+ cells (40), which plays an important role in the course of psoriasis (41), underlie new paradoxical strategies for the treatment of diseases, in which the therapeutic effect is determined by a compensatory response, rather than drug effect (42). Paradoxical strategies for the treatment of diseases can be applied with the use of drugs that affect signaling pathways, triggering cascades of biochemical processes both in the cell and throughout the body. Accounting for the whole diversity of signaling pathways becomes possible with the use of systems biomedicine methods that are increasingly being introduced into medical practice (43, 44) and contribute to improving the effectiveness of treatment on the base of a precise ttreatment.

## Conclusion

The carried out investigation have shown high therapeutic efficiency and safety of application of muramyl peptide at psoriasis. Based on the results of the conducted studies with full justification, it is possible to recommend the use of the muramyl peptide GMDP in all cases of plaque psoriasis at various stages of its development. It is inappropriate to use it in patients with psoriasis in cases of the emergence of immunodeficiency states after repeated use of immunosuppressive therapy.

The pathogenetic efficacy of muramyl peptide in patients with psoriasis is provided by its ability to normalize the balance of immunocompetent cells, cytokines, adhesion molecules, and nitric oxide, involved in the realization of inflammation. The impact of glucosaminyl muramyl dipeptide in the treatment of psoriasis could be of potential therapeutic benefit as it influence on the pathways of maintaining immune homeostasis evolutionary formed during coexistence of commensal microflora and host.

## Author Contributions

SG: idea. VU and AK: design and data collection. SG, VU, and AK: analysis and manuscript writing.

### Conflict of Interest Statement

SG was employed by the company Peptek. The remaining authors declare that the research was conducted in the absence of any commercial or financial relationships that could be construed as a potential conflict of interest.
